# Physicians’ perception of generic and electronic prescribing: A descriptive study from Jordan

**DOI:** 10.1186/2052-3211-7-7

**Published:** 2014-06-19

**Authors:** Faris El-Dahiyat, Reem Kayyali, Penelope Bidgood

**Affiliations:** 1 Faculty of Pharmacy, Isra University, Amman, Jordan; 2 Pharmacy Department, Kingston University, Penrhyn Road, Kingston upon Thames KT1 2EE, UK; 3 Mathematics Department, Kingston University, Penrhyn Road, Kingston upon Thames KT1 2EE, UK

**Keywords:** Generic medicines, Generic substitution, Electronic Prescribing, INN prescribing, Policy, Physicians

## Abstract

**Objectives:**

The aim of this study was to investigate Jordanian physicians’ perception and attitudes toward generic medicines and generic substitution. It also aimed to examine factors that affect physicians’ pattern of prescribing, and to evaluate their opinion regarding future introduction of Electronic Prescribing (EP) in Jordan.

**Methods:**

A cross-sectional descriptive study involving Jordanian physicians working in both public and private sectors was undertaken, using a self-administrated anonymous questionnaire. Frequency tables, cross-tabulation and chi square tests were used for data analysis. The response rate was 75.2% (n = 376/500).

**Results:**

Cost was claimed to be an important factor in the prescribing decision for 69.1% of the Jordanian physicians. The majority of physicians (77.4%) claimed that they often prescribe generic medicines. Jordanian physicians predominantly welcomed the implementation of an EP and International Nonproprietary Name (INN) prescribing systems with 92%, and 80.1% respectively. More than two thirds of the physicians (69.4%) accepted generic substitution by pharmacists, with a significant association with their employment sector; physicians who work in the private sector tended to oppose generic substitution compared with physicians who work in the public sector. Physicians mostly (72.1%) opposed that generic substitution should only be allowed upon patient request.

**Conclusions:**

Jordanian physicians have a positive attitude towards generic medications and high willingness and acceptance of strategies that encourage generic utilisation such as EP, INN prescribing and generic substitution. All these strategies would help reduce the high expenditure on medicines in Jordan. These findings would provide baseline data to policy makers to develop a robust generic policy to achieve greater clinical effectiveness and economic efficiency from medicines prescribing.

## Introduction

The high health care expenditure on pharmaceutical products is becoming a challenging issue worldwide [1,2]. In 2007, the expenditure on drugs in Jordan exceeded US$ 700 million, which accounted for around one-third of the national health care budget. Moreover, these costs are believed to be higher than most countries that have similar income calibre of Jordan [3].

In general, generic medicines are 20% to 90% cheaper than the innovator medicine, and their utilisation represents a well-established strategy for controlling health care expenditures [4-6]. A generic medicine is defined as a medicinal product which is identical in its active ingredient qualitative and quantitative composition, and is bioequivalent to an originator medicine, whose granted patent protection has expired [7,8]. Generic medicines are generally marketed under the non-proprietary name or could be marketed as branded generics [9], as in the case of Jordan where 97% of generic medicines are branded [10]. Public and private third party payers and health care authorities increasingly encourage or mandate the use of generics through measures such as generic prescribing and generic substitution [11-15]. In 2002, a circular from the Jordanian Ministry of Health required doctors in public hospitals and health clinics to prescribe generically [16].

The prescribing behaviour of physicians is considered to be crucial for generic utilisation as they determine whether their patients need originator drugs or generic drugs [17]. A generic medicine may not always be suitable for the patient [18]. Several factors may play a significant role in influencing the physicians ‘prescribing behaviour such as the “trust” and the “quality image” of the pharmaceutical company [19]. Physicians’ prescribing behaviour can also be influenced by pharmaceutical companies through a variety of incentives such as high-end education programs or even some cash payment for prescriptions [20]. In addition, free samples and gifts that include financing for domestic and international conference participation, travel and accommodation, medical education, meals, honoraria and small gifts like pens can also influence prescribing. However, one cannot state that physicians prescribe only on the basis of the rewards that they receive from the company, but the rewards certainly help physicians to remember the company brands [21,22]. Therefore, these incentives may indirectly affect the patients, by encouraging them to use higher priced originator products instead of equally effective, lower-cost generics [23].

Patients’ requests and preference play a vital role in prescribing behaviour and according to previous research when physicians do not comply with patient requests, patients are less satisfied with their physician visit [24,25].

Globally, physicians are much more sensitive to arguments about a drug's efficacy than about its price [26,27]. The effect of price and cost of medicine was found to be insignificant on physician prescribing behaviour [28]. As they do not bear the full cost of the prescribed drug, or they possess limited information about cost and prices of medicines [29-31].

An efficient source of information about the cost of medicines is believed to be through E-prescribing system (EP), where prescriptions are generated within e-prescribing systems and are transmitted electronically to pharmacies through a secure network between physician office and community pharmacies [32]. This involves direct computer-to-computer transmission of prescriptions [33]. EP not only reduce health care costs by avoiding adverse drug events and substitution to less expensive medicine, but also enables the prescribers to check patients’ health plan or insurance coverage at the point of care. Additionally it offers physicians a powerful tool to manage their patients’ medication in a safe and efficient way. EP can enhance patient safety and medication compliance , improve prescribing accuracy and efficiency, decrease pharmacy costs, reduce phone calls between pharmacists and physicians, reduce data entry, expedite prescription refill requests compared to paper-based prescribing, and eliminate handwriting interpretation errors [34,35].

It was reported that 7000 patients die every year in the US due to medication error [36], including error caused by illegible handwritten prescriptions. As a result, the use of EP was promoted [37]. In another study which was conducted in a UK hospital, there was a significant reduction in both pharmacists' interventions and prescribing errors following the introduction of EP. Interventions were reduced from 3.0% on all medication orders to 1.9%, and errors from 3.8%to 2.0% [38]. Moreover, a previous study found that physicians using an EP system increased their generic substitution rate by 15% and increased generic prescribing by more than 8% [39].

In Jordan, despite the continuous increase in pharmaceutical expenditure, a pharmaceutical policy focusing on the promotion of generics utilisation has never been developed. Therefore, the aim of this paper was to investigate physician perception and attitudes toward generic medicines and generic substitution, to examine factors that affect this pattern of prescription, and to evaluate their opinion regarding future introduction of EP in Jordan. The findings from this study would provide a baseline data for the introduction of a robust generic policy and eventually the use of more efficient measures to control pharmaceutical expenditures.

## Methods

In this cross sectional study a questionnaire was carried out to collect data from Jordanian physicians working in private or public sectors, as physicians who are working in the public sectors are not allowed to work private sector, in order to measure physicians’ prescribing behaviour, and their perceptions towards generic medicines and issues pertaining the use of generics in Jordan.

Anonymity of respondents was preserved in the study, as names of participants were not included.

The questionnaire was tested for face and content validity by two experts. The wording of the survey was further revised after pilot testing with ten physicians. Moreover, some questions were presented and explained in better way. There are four sections in the questionnaire. The first section evaluates the prescribing behaviour of the responding physicians. The following section was exploring physicians’ perception towards generic medicines. The third section measures physicians’ opinion regarding issues pertaining the use of generics in and the introduction of EP in Jordan. The last section characterised the respondents’ demographics. The responses were framed in different type such as single, multiple (participants were allowed to choose more than one answer) and four point likert scale (1 = strongly disagree, 2 = disagree, 3 = agree and 4 = strongly agree).

According to the Jordanian Medical Association, the entire sample population is 17000 physicians; From the 500 questionnaires which were distributed, 376 questionnaires were collected from physicians in private and public sectors and included in this study which gives a response rate of 75.2%. This is a representative sample from the population (N = 17000) based on 5% margin of error and 95% confidence level.

The participation of physicians was strictly voluntary. The informed consent of the participants was obtained and no personal data of the participants were reported. Data was collected from 2nd June 2012 to 15st July 2012. All the collected data was entered into PASW^®^ 18.0 for descriptive analysis using frequency and cross-tabulation and chi square tests. This study was approved by the Research Ethics Committee of Kingston University, London.

## Results

### Demographic characteristics of responding physicians

A total of 376 responses were included, the basic demographic of the responding physicians is summarised in Table 1. The sample was distributed between male (240, 63.8%) and female (136, 36.2%). The modal age of the responding physicians were between 30 years and 40 years. Respondents had different years of experience in practicing medicine; the modal years of experience were from 6–10 years. Regarding the employment sector, almost the same number of responses was collected from physician working in private and public sectors (Table 1).

**Table 1:** Demographics and practice characteristics

***Characteristic***	***N (%)***
***Gender***	
Male	240 (63.8)
Female	136 (36.2)
***Age group, (years)***	
Under 30	91 (24.2)
30-40	135 (35.9)
41-50	105 (27.9)
51-60	35 (9.3)
Above 60	10 (2.7)
***Practicing, (years)***	
1-5	96 (25.2)
6-10	100 (26.6)
11-15	75 (19.9)
16-20	70 (18.6)
21 and above	35 (9.3)
***Employment Sector***	
Private	180 (47.9)
Public	196 (52.1)

### Prescribing behaviour

**Figure 1:**
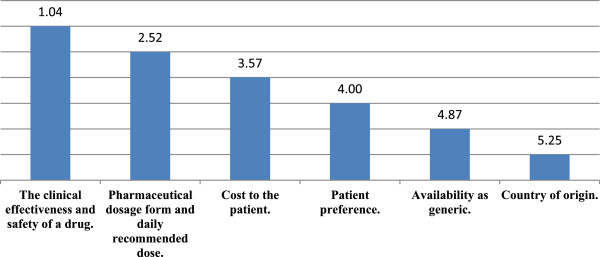
Rankings of the means for factors that in fluence prescribing behaviour of Jordanian physicians.

When assessing the rank of the factors that may influence physicians’ decision when prescribing a medicine, the first factor was the clinical effectiveness and safety of a medicine prescribed with a mean of 1.04. The second factor was the dosage form and daily recommended dose with a mean rank of 2.52, the cost of medicine was the third factor affecting physicians decision with a rank of 3.57, the forth factor was patient preference with a mean rank of 4.00. However, the fifth rank was availability as a generic and the sixth rank was for country of origin of a medicine with means of 4.87 and 5.25 respectively (Figure 1).

The physicians’ prescribing behaviour was evaluated, the majority of the respondents (86.7%) use international treatment guidelines to justify their prescribing decision. An almost equal percentage (57.4% and 54.5%) use local guideline and local protocols or medical journals publication and online databases respectively. Conferences and pharmaceutical sales representatives were used by 37.2% and 12% of the physician respectively in order to justify their prescribing decision. Few responders (2.7%) justify their decision by other reasons such as their own experience and patient clinical history.

### Cost of medicine

The physicians were asked about the importance of cost in their prescribing decision, 58.5% of them believed that the cost is important, 10.6% believed that the cost is highly important, whereas 30.9% of the physicians believed that the cost is not important at all.

Further analysis showed that the community pharmacists were the main source for physicians in order to get the information about cost of medicine as mentioned by 77.1% of the responders. The second source used by 65.4% of responding physician was pharmaceutical sale representatives, while Jordan food and drug administration (JFDA) website was used only by 20.2% of physicians. Other source used was the patients themselves according to 9.3% of responders.

### Current generic prescribing

When assessing how often physicians prescribe generic medicine instead of originator brand in their current practice, only 1.3% of the participants stated hardly ever and 21.3 stated % rarely. However, 62.8% of the physicians often prescribe generic and 14.6% of the physicians very often prescribe generic medicine instead of an originator brand. A chi-square statistic was calculated to examine if there is a relation between the employment sector of the responders and whether or not they prescribe generic medicines in their daily practice. The test was found to be statistically significant; the value of chi square is 54.580 with a P value < 0.05. Physicians working in public sectors are more likely to prescribe generic medicines.

When physicians were asked about how often they write their prescriptions using the International Non-priority Name (INN), only 4% of the responders stated very often. An equal percentage (43.9%) used INN either often or rarely, and 8.2% hardly ever used INN.

There was a significant correlation between physicians’ employment sector and whether or not they write their prescription using the INN. The value of chi square is 28.195 with a P value < 0.05. Physicians working in public sectors are more likely to prescribe using INN.

### Perceptions about generic substitution

When assessing the physicians’ perception on generic substitution, 96% of responding physicians agreed that the ability to perform generic substitution will ensure prompt availability of medications to the patient and that generic substitution will increase the use of locally produced medicines. Further analysis found that 92.1% of the physicians perceived that generic substitution offer significant cost advantage to the patient. In addition, 74.7% believed that such a practice will allow pharmacists to select the most affordable drug to a patient (Table 2).

**Table 2:** Jordanian physicians ’ responses to four point likert scale questions exploring perceptions towards generic medicines and issues pertaining the use of generics in Jordan

**Survey questions/Statement**	**Frequency (%)**			
	**Strongly disagree**	**Disagree**	**Agree**	**Strongly agree**
Generic substitutions will increase the use of locally produced medicines.	5 (1.3%)	10 (2.7%)	276 (73.4%)	85 (22.6%)
Ability to perform generic substitution will ensure prompt availability of medications to the patient	0 (0.0%)	15 (4.0%)	216 (78.7%)	65 (17.3%)
Generic substitution offer significant cost advantage to the patient.	0 (0.0%)	30 (8.0%)	271 (72.1%)	75 (19.9%)
Generic substitution will allow pharmacists to select to select the most affordable drug to a patient.	5 (1.3)	90 (23.9%)	256 (68.1%)	25 (6.6%)
Developing a computerized system which includes important information about drugs such as: medicines interaction, contraindications and cost, would improve the prescribing process	0 (0.0%)	5 (1.3%)	180 (47.9%)	191 (50.8%)
Implementing an electronic prescription service would result in a more efficient prescribing and dispensing process.	0 (0%)	30 (8.0%)	241 (64.1%)	105 (27.9%)
Standard guidelines on generic substitution process to both physicians and pharmacists should be implemented.	0 (0.0%)	10 (2.7%)	291 (77.4%)	75 (19.9%)
Quality use of generic medicines among Jordanian consumers can be achieved if both physicians and pharmacist work together.	0 (0.0%)	35 (9.3%)	256 (68.1%)	85 (22.6%)
It is feasible to implement prescribing system based on International Non-priority Name (INN).	5 (1.3%)	70 (18.6%)	241 (64.1%)	60 (16.0%)
Community Pharmacist in Jordan should be given generic substitution right.	25 (6.6%)	120 (31.9%)	160 (42.6%)	71 (18.9%)
Generic substitution should be allowed only at patient request.	80 (21.3%)	191 (50.8%)	85 (22.6%)	20 (5.3%)

**Figure 2:**
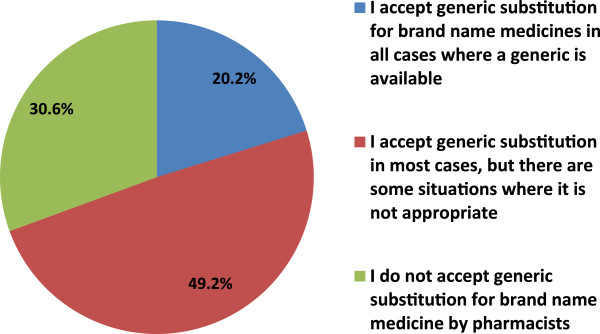
General opinion regarding generic substitution by community pharmacists.

**Figure 3:**
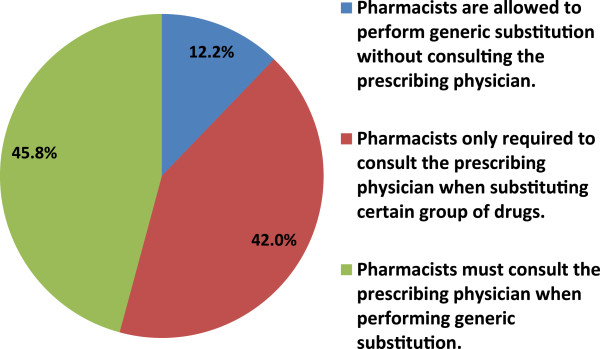
Generic substitution preferred practice according to physicians who accepted it in most or all cases.

Giving community pharmacists in Jordan generic substitution right was agreed by 61.5% of the responders. On the other hand, 72.1% of the physicians opposed that generic substitutions practice should be allowed upon patient request only (Table 2).When assessing physicians’ general opinion regarding generic substitution by community pharmacists, around half of them (49.2%) accepted generic substitution in most cases as there are some situations where it is not appropriate and 20.2% accepted it in all cases where a generic is available, Interestingly, 30.6% do not accept generic substitution by pharmacists at all (Figure 2). There was a significant correlation between physician’ employment sector and whether or not they accept generic substitution. The value of chi squares was 11.87 with a P value < 0.05. Physicians working in public sector tended to accept generic substitution more compared with physicians working in private sector.When physicians who accepted the generic substitution in the previous question either in most or in all cases were asked about preferred generic substitution practice, 45.8% of them believed that pharmacists must consult them when performing generic substitution. However, 42% of the responders preferred that pharmacists only consulted them if they are substituting certain group of drugs (e.g., narrow therapeutic index). Only 12.2% of the physicians who accepted generic substitution in most or all cases believed that pharmacists should be allowed to perform generic substitution without consulting the prescribing physician (Figure 3). There was a significant correlation between physicians’ employment sector and the standard of practice, the value of chi squares is 10.85 with a P value <0.05. By reviewing the cross table, physicians working in public sector believe that pharmacists should allowed to perform generic substitution without consulting them.

### Perceptions regarding future introduction of EP in Jordan

Nearly all of the responding physicians (98.7%) agreed that developing a computerised system which includes important information about drugs such as: medicines interaction, contraindications and cost, would improve the prescribing process. The implementation of an EP system would result in a more efficient prescribing and dispensing process according to 92% of the responders (Table 2).

However, majority of physicians (80.1%) agreed to the implementation of a prescribing system based on INN (Table 2).

The majority (97.3) also believed that standard guidelines on generic substitution for both physicians and pharmacists should be implemented. Furthermore, 90.7% agreed that quality use of generic medicines among Jordanian consumers can be achieved if both physicians and pharmacists work together.

## Discussion

The purpose of this study is multifaceted. We wanted to investigate physicians’ opinions about substituting generic medications for brand name drugs and their prescribing behaviour and opinions regarding the introduction of an EP system.

In general, physicians were concerned about the efficacy of a drug rather than its price when making prescribing decisions [40,29]. In the current study, clinical effectiveness was the most important factor that influenced the prescribing behaviour of physicians in Jordan. The second factor was the pharmaceutical dosage form and recommended daily defined dose, with the cost of the prescribed medicine being the third important factor. These results are consistent with the results of a previous study that measured the prescribing behavior of physician in Greece and Cyprus [41].

Previous studies considered the cost as an important factor in physicians prescribing behaviour. In a study that was held in America, the cost was an important factor when prescribing especially for uninsured patients [42]. Additionally, results from a qualitative study in Denmark showed that drug cost was considered an important factor influencing prescribing decisions [5]. Moreover, a study in Greece and Cyprus found that 60% of the physicians consider the cost as important [41]. In this study the views of physicians were consistent with these studies, 69.1% of them claimed that the cost is important in their prescribing decision. There was a significant association between the consideration of the cost while prescribing and physician’s employment sector. Physician working in the public sector were more likely to consider the cost when prescribing than their counterparts in the private sector.

From the findings, physicians use international treatment guidelines as well as local guideline and local protocols as main sources to justify their prescribing decisions. Medical journals publication and online databases come after and then conferences. However, pharmaceutical sales representatives were claimed to be of least importance. This contradicted the previous studies in which pharmaceuticals sale representatives were more important sources of information in New Zealand [43], Denmark [44] and in Nigeria [45].

Only 9.3 % of the physician reported that patient communication was the source of medicines’ cost. Patients hardly ever communicate with their physicians about medication choices and out-of-pocket costs of medications [46,47].

One of the most important findings of this study is that physicians seem to be open to prescribing generic medicines; the majority of the responders 77.4% claimed that they often prescribe generic medicines. However, only 47.9% of the Jordanian physicians claimed to be writing their prescriptions currently using the International Non-priority Name (INN). This variation in percentage could be due to the fact that about (97%) of the locally produced medicines are branded generic [10]. This indicates, however, that physicians in Jordan hold a positive view about generic medicines. This was similar to a study in Malaysia where the majority of the physicians (85.1%) claimed that they actively prescribed generic medicines in their practice [48]. On the other hand, in Greece, only one of four physicians (24.8%) prescribed generic medicines [49].

This study found that if a prescribing system based on the INN was implemented, 80.1% of the physicians are willing to use it. This was similar to a French study, where the majority of physicians (76.2%) declared that they were willing to write their prescriptions using INN [50]. Using an INN prescribing system not only would minimise confusion but also would improve patient acceptability of generic medicines.

Almost all Jordanian physicians believed that developing a computerised EP system which includes important information about drugs such as; medicines interaction, contraindications and cost, would improve the prescribing process and result in a more efficient prescribing and dispensing process. Implementing such a prescribing system not only would support improved medication adherence [51], but also reduce cost through generic utilisation.

Majority of Jordanian physicians (49.2%) accepted generic substitution in most cases as there are some situations where it is not appropriate (e.g. for narrow therapeutic index drugs) and 20.2% accepted it in all cases where a generic is available. Whereas, 30.6% did not accept generic substitution by pharmacists at all. On the other hand, the results from a previous study in America showed that 78% of physician supported generic substitution in most cases, 17 % supported the substitution in all cases where generic is available and only 5% do not support substitution at all [52].

Physicians mostly (72.1%) opposed that generic substitution should only be allowed upon patient request. Despite the widespread belief that medical decisions are sensitive to patients' expectations [53], the choice of prescribed drugs appears to result essentially from the physician's own decision-making process [54]. Nevertheless, 61.5% agreed to give the pharmacist the substitution right.

It was observed statistically that there is a significant association between physicians’ acceptability for generic substitution and their employment sector; physicians who work in the private sector tended to oppose generic substitution compared with physicians who work in the public sector. This finding was similar to previous studies in which private physicians were 50–80% more likely to oppose substitution, as they might have stronger brand-name loyalty. This could be due to private sector physicians being less restricted to participate in educations and conferences paid for by pharmaceutical firms, or to perform paid assignments for them compared to public physicians as there is many rules restricting them from such participation [55]. Therefore, private physicians’ prescribing behaviour may be influenced by pharmaceutical companies through a variety of incentives such as high-end education programs or even some cash payment for prescriptions [20]. These incentives may indirectly affect the patients, by encouraging them to use higher priced originator products instead of equally effective, lower-cost generics [23].

There are many benefits for generic substitution to be implemented in Jordan. It will ensure prompt availability of medications to the patients, and it will support the local industry by increasing the use of locally produced medicines. Generic substitution will also offer significant cost advantage to the patient by allowing the selection of the most affordable drug to a patient.

The finding from this study suggested that, in order to increase the generic utilisation in Jordan, standard guidelines on generic substitution process to both physicians and pharmacists should be implemented. Furthermore, the results highlighted that the quality use of generic medicines among Jordanian patients can be achieved if both physicians and pharmacists worked together.

Jordanian physicians had stated that there is a need for a standard guideline on generic substitution. The adoption of a standard guideline for both physicians and pharmacists on how and when to perform generic substitution for their patients or by introducing legislation for compulsory generic substitution wherever appropriate would further encourage the use of generic medicines and maintain accessibility and affordability of medicines^.^[50,56].

## Conclusion

The findings from this study showed the positive attitude of Jordanian physicians towards generic medications and their high willingness and acceptance of strategies that encourage generic utilisation in Jordan such as generic substitution, INN prescribing and EP. All these strategies would help reduce the high expenditure on drugs in Jordan which accounted for around one-third of the national health care budget [3].

These insights will help policy makers in Jordan to develop a robust generic policy which could be used to achieve to greater clinical effectiveness and economic efficiency from drug prescribing.
